# Sperm length divergence as a potential prezygotic barrier in a passerine hybrid zone

**DOI:** 10.1002/ece3.7768

**Published:** 2021-06-16

**Authors:** Emily R. A. Cramer, Gaute Grønstøl, Logan Maxwell, Adrienne I. Kovach, Jan T. Lifjeld

**Affiliations:** ^1^ Natural History Museum University of Oslo Oslo Norway; ^2^ Natural Resources and the Environment University of New Hampshire Durham NH USA

**Keywords:** hybridization, phenotypic divergence, postmating prezygotic barriers, speciation, sperm morphology

## Abstract

The saltmarsh sparrow *Ammospiza caudacuta* and Nelson's sparrow *A. nelsoni* differ in ecological niche, mating behavior, and plumage, but they hybridize where their breeding distributions overlap. In this advanced hybrid zone, past interbreeding and current backcrossing result in substantial genomic introgression in both directions, although few hybrids are currently produced in most locations. However, because both species are nonterritorial and have only brief male–female interactions, it is difficult to determine to what extent assortative mating explains the low frequency of hybrid offspring. Since females often copulate with multiple males, a role of sperm as a postcopulatory prezygotic barrier appears plausible. Here, we show that sperm length differs between the two species in the hybrid zone, with low among‐male variation consistent with strong postcopulatory sexual selection on sperm cells. We hypothesize that divergence in sperm length may constitute a reproductive barrier between species, as sperm length co‐evolves with the size of specialized female sperm storage tubules. Sperm does not appear to act as a postzygotic barrier, as sperm from hybrids was unexceptional.

## Graphical abstract

Sperm length differs between saltmarsh and Nelson's sparrows, two species that hybridize, and length correlates with both hybrid index (based on 135 SNPs) and plumage index. This divergence in sperm phenotype may act as a reproductive barrier between species.

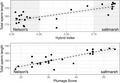

## 1 INTRODUCTION

Allopatric populations may evolve divergent phenotypes due to differing ecological pressures and sexual selection, potentially resulting in pre‐ and postzygotic reproductive isolation upon secondary contact (Coyne & Orr, 2004). Determining which phenotypes have diverged and how diverged phenotypes contribute to reproductive isolation upon secondary contact is an integral part of understanding speciation (Shaw & Mullen, 2011), for example, complementing the understanding of how genomic architecture impacts speciation (Campbell et al., 2018) and how selection on one isolating mechanism impacts the evolution of other reproductive barriers (Lorch & Servedio, 2007).

Adaptation to divergent ecological pressures appears to have played a key role in speciation between the sister species saltmarsh sparrow (*Ammospiza caudacuta*) and Nelson's sparrow (*A. nelsoni*) (e.g., Walsh et al., 2018, 2019; Walsh, Olsen et al., 2016). The saltmarsh sparrow is restricted to coastal marshes, while Nelson's sparrow inhabits a broader range of habitats, including inland marshes and coastal marshes with lower salinity (Greenlaw, 1993). Genes involved in plumage melanism and osmotic balance show elevated differentiation between the populations and are thought to be adaptive in the respective environments (Walsh et al., 2019). The two species hybridize along a narrow stretch of coast in North America, with high genetic admixture despite the rarity of intermediates equivalent to F1 hybrids (Maxwell et al., 2021; Walsh, Kovach et al., 2018; Walsh et al., 2015). Within the hybrid zone, several genes involved in osmotic balance show particularly high introgression, which is hypothesized to be adaptive in the context of the mosaic of habitats, with varying salinity, found within the hybrid zone (Walsh, Kovach et al., 2018). Postzygotic isolation appears limited, as hybrids of both sexes produce offspring (Walsh et al., 2018; Walsh, Olsen et al., 2016). However, evidence suggests a reduction in female hybrid survival from nestling to adult (Maxwell et al., 2021; Walsh, Olsen et al., 2016), consistent with Haldane's rule, which predicts selection against hybrids of the heterogametic sex due to genetic incompatibilities or other endogenous factors (Haldane, 1922).

In addition to ecological adaptation, the species have divergent mating phenotypes and behaviors which may contribute to prezygotic isolation via sexual selection (Walsh, Maxwell et al., 2018). Nelson's sparrows are smaller with less‐defined plumage streaking (Greenlaw, 1993; Walsh et al., 2015). Nelson's sparrow males sing, perform song flights, and guard females for a short period after copulation (Greenlaw, 1993; Shriver et al., 2007). In saltmarsh sparrows, such courtship is not seen, and instead, groups of males frequently chase a female together and attempt copulation, without guarding, suggesting substantial opportunity for sperm competition and cryptic female choice (Greenlaw, 1993; Shriver et al., 2007). Females may also incite copulations and thereby competition among males (Greenlaw & Post, 2012). Anecdotal evidence suggests that saltmarsh sparrow males preferentially follow and copulate with conspecific females (Greenlaw, 1993). Potentially due to these phenotypic differences, offspring production occurs primarily within species at coastal sites throughout the hybrid zone, with more conspecific offspring produced than would be expected via random mating, given the proportion of available partners of each species (Maxwell et al., 2021; Walsh, Maxwell et al., 2018). However, because male–female interactions are brief and the species are not territorial (Shriver et al., 2010), inferences of assortative mating are based on the genetic parentage of offspring rather than direct observations (Walsh, Maxwell et al., 2018). Inferring copulation patterns from parentage data is, however, not straightforward for species with sperm competition, since genetic parentage depends on successful fertilization of eggs as well as on copulations (Cramer, 2021). Further work is therefore needed to understand whether sperm and female reproductive phenotypes have also diverged, since poor sperm performance following heterospecific copulation can cause reproductive barriers between species (Howard et al., 2009).

Two lines of evidence suggest that sperm phenotypes are likely to have diverged between these species. First, females of both species copulate with multiple males in each nesting cycle, as evidenced by the high proportion of broods sired by more than one male (estimated for saltmarsh sparrows at 88% of 112 broods, Walsh, Maxwell et al., 2018, 79% of 48 broods, Maxwell, 2018, and 95% of 60 broods, Hill et al., 2010; for Nelson's sparrows 100% of 14 nests, Walsh, Maxwell et al., 2018, and 81% of 26 broods, Maxwell, 2018). This high level of multiple mating generates opportunity for postcopulatory sexual selection on sperm phenotypes via sperm competition and cryptic female choice, and frequent multiple mating is associated with rapid evolution of sperm morphology in other passerine birds (Rowe et al., 2015). Sexual conflict in saltmarsh sparrows may also promote cryptic female choice, because females may occasionally accept copulations from unpreferred males to avoid prolonged harassment (Greenlaw & Post, 2012). Consistent with high levels of sperm competition and/or cryptic female choice, both species have relatively large testis volumes (Rising, 1996) and the saltmarsh sparrow has a large cloacal protuberance (i.e., where sperm is stored prior to ejaculation), which may reflect high sperm availability (562.8 ± 12.39 mm^3^, Greenlaw & Post, 2012; expected value for their body size, approximately 200 mm^3^ from fig 3 in Briskie, 1993). Secondly, several genes with sperm‐related function (MSMB, OVGP1, and CCNI) show significant divergence between the species (Walsh et al., 2019). However, divergence between species in sperm phenotype has not been examined.

In this study, we characterized sperm morphology in the saltmarsh–Nelson's sparrow hybrid zone, as a first step in assessing the possible role of sperm as a pre‐ and postzygotic barrier. We hypothesized that sperm morphology has diverged between the species and that among‐male variation in sperm morphology should be low, since species in which females copulate frequently with multiple males show relatively uniform sperm morphology (Lifjeld et al., 2019). We evaluated two aspects of sperm phenotype that are particularly relevant as possible prezygotic barriers: total sperm length and sperm head morphology. Total sperm length may affect sperm‐female interactions, since females store sperm in specialized sperm storage tubules prior to using them to fertilize eggs (Bakst et al., 1994). Further, total sperm length correlates with tubule length across species (Briskie et al., 1997), suggesting a need for compatibility in length between sperm and female sperm storage tubules. The sperm head is the portion of the cell that interacts most directly with the egg, including undergoing the acrosome reaction at the point of fertilization (Nishio & Matsuda, 2017), and sperm head morphology shows character displacement in another passerine hybrid zone (i.e., elevated differentiation within the hybrid zone, compared to outside it; Albrecht et al., 2019). In addition to comparing sperm morphology between species, we evaluated how sperm morphology related to a genetic hybrid index and a plumage index reflecting plumage features diagnostic for the species, to better contextualize the evolutionary potential and selective pressures on sperm morphology, respectively. To assess the potential for sperm to act as a postzygotic barrier, we characterized the morphology of sperm from hybrid males. A broad range of hybrid sperm phenotypes is observed in other hybridizing passerine sister (or near‐sister) species pairs, ranging from an absence of normal sperm cells (Ålund et al., 2013) to apparently normal morphology and swimming performance (Albrecht et al., 2019; Cramer et al., 2015). Given that hybrid males produce offspring in this hybrid zone (Maxwell et al., 2021; Walsh, Maxwell et al., 2018), we expected to find some normal sperm cells among hybrid males.

## 2 MATERIAL AND METHODS

### 2.1 Field procedures

Sparrows were sampled during the 2016 breeding season (May–August), as part of a larger study (Maxwell et al., 2021) at two locations in Mid‐Coast Maine, USA, near the center of the hybrid zone: Popham Beach State Park (43.739, −69.806) and Maquoit Bay (43.867, −69.988). Both sites exhibit relatively equal species abundances and show high levels of introgression and backcrossing in both directions (Maxwell et al., 2021).

Birds were captured through mist netting and banded with a USGS aluminum leg band and a single plastic color band to denote sampling location. Blood was collected from the brachial vein (10–20 µl), transferred to Nobuto Filter Paper (Sterlitech, Kent, Washington), and stored at room temperature until analysis. We attempted to collect ejaculates via cloacal massage from adult males; fluid was obtained from 46 males (21 saltmarsh, 19 Nelson's, and 6 intermediates), with additional males not producing any fluid. After mixing ejaculate samples with 10–20 µl PBS, samples were transferred to 10% formalin for storage.

Individuals’ plumage phenotypes were assessed in the field by visually scoring 13 plumage traits on a range from 1 to 5, with lower number representative of Nelson's sparrows and higher numbers representative of saltmarsh sparrows (Shriver et al., 2005; Walsh et al., 2015). A sum of all the scores typically allows for an assignment to closest parental species, but does not reliably allow for distinguishing pure from back‐crossed individuals of either species, nor for distinguishing hybrids from other categories (Shriver et al., 2005; Walsh et al., 2015). For two males, plumage index data were not available.

### 2.2 Genomic analysis

To determine the genotype of each sparrow, we performed double digest restriction‐site‐associated DNA (ddRAD) sequencing, after digesting genomic DNA with the restriction enzymes SbfI and MspI. We followed the Peterson et al. (2012) protocol, as described in Maxwell et al. (2021). Thirty allopatric individuals of each parental species were also included as reference for building a hybrid index (Maxwell et al., 2021). Allopatric Nelson's sparrow sample locations included Upper Narraguagus and Hobart Stream in Maine, as well as Wolfville and Yarmouth, Nova Scotia, Canada. Allopatric saltmarsh sparrow locations included Sawmill Creek, Idlewild Park, Marine Nature Center, and Shirley, New York; Sachuset, Rhode Island; Barn Island, Connecticut. Dual‐indexed ddRAD libraries were sequenced at the Cornell University Institute for Biotechnology across three Illumina HiSeq 2,500 lanes and one HiSeq 2,500 rapid run lanes of (100 bp reads).

Raw sequences were assessed for overall quality using fastQC (Andrews, 2010) and subsequently trimmed and filtered using FASTX‐Toolkit (Hannon, 2010). We trimmed reads on the 3’ end to 97 bp and eliminated reads that had Phred quality scores less than 10 and those for which 95% of the bases had scores less than 20. Reads were demultiplexed and filtered for completeness and Illumina's chastity/purity scores using STACKS v 1.48. We discarded reads that did not meet chastity/purity filters, that had an uncalled base(s), that had greater than 1 mismatch in the adapter sequence, or that did not include an intact SbfI RAD cut site and one unique barcode. Resulting sequences were trimmed (fastx_trimmer) to the length of the shortest read and aligned to the saltmarsh sparrow genome (Walsh et al., 2019) using the end‐to‐end option in Bowtie 2 v 2.2.9. STACKS v 1.48 was subsequently used to build a catalog of SNPs, with a minimum stack depth of 6, and no more than 5 mismatches allowed between sample loci. We further filtered catalog loci based on the mean log likelihood of the locus in the population (−300), which resulted in a total of 5,391 SNPs. Finally, we created a subset of SNPs across all individuals to be used in identifying fixed differences between the species and developing a hybrid index. We chose one SNP per locus, which was typed in at least 50% of the population at a minimum stack depth of 6. We grouped all individuals into an admixed and two allopatric populations and calculated the fixation index (*F*_st_) for each SNP using VCFtools (Danecek et al., 2011). We identified 135 SNPs that were fixed between species (*F*_st_ = 1), and we subsequently used these SNPs to calculate a hybrid index and determine the genotype of each sparrow.

We calculated a hybrid index indicating the proportion of saltmarsh sparrow alleles (0 = pure Nelson's sparrow, 1 = pure saltmarsh sparrow) and interspecific heterozygosity as the proportion of these 135 species‐specific markers that were heterozygous (package Introgress, Gompert & Buerkle, 2010). We here considered three categories: pure and/or back‐crossed Nelson's sparrows (hybrid index <0.25 and interspecific heterozygosity <0.3); pure and/or back‐crossed saltmarsh sparrows (hybrid index >0.75 and interspecific heterozygosity <0.3); and intermediates (hybrid index between 0.25 and 0.75, with interspecific heterozygosity >0.3; this category includes F1 and F2 hybrids). These designations follow the logic of Milne and Abbott (2008) in inferring individuals’ ancestry from their genotypes, and they follow earlier studies on this system (Maxwell et al., 2021; Walsh et al., 2016; Walsh et al., 2016). These assignments were used in comparisons across species. For two males that did not produce sperm following cloacal massage, genetic information was not available.

### 2.3 Sperm analysis

To measure sperm morphology, approximately 15 µl fixed ejaculate was streaked onto a microscope slide, air‐dried, and rinsed with distilled water. Up to 10 haphazardly chosen, morphologically normal sperm cells were photographed with a camera mounted on a digital light microscope (320X magnification, Leica DM6000B and DC420) and measured to the nearest pixel (0.14 µm) using Leica Application Suite v. 4.1.0 (Leica Microsystems, Switzerland). For four males with fewer than 10 cells on the first microscope slide, an additional slide was made with 30 µl fixed ejaculate; additional slides were not considered for other males due to sample evaporation. The length of the head (including acrosome), the midpiece (which consists of a single fused mitochondrion that wraps around much of the length of the flagellum), and the tail (exposed flagellum not wrapped by midpiece) were measured following Kleven et al. (2008). We assessed measurement repeatability by remeasuring all 10 sperm cells for each of 5 saltmarsh and 5 Nelson's sparrow males, with repeated measurements taken blindly with respect to the original measurements, four years apart. Repeatability, or the percent of variance attributable to the random effect of sperm cell identity, was high and significant, in models that controlled for male identity as a fixed effect (Nakagawa & Schielzeth, 2010; Stoffel et al., 2017): head: 89.0%, midpiece: 98.3%, tail, 95.4%; all *p* < .001. Total sperm length was calculated as the sum of these three segments. The within‐ejaculate variation in total sperm length (CV_wm_) was calculated for males with ≥5 cells measured. Using the mean total sperm length for each male, we also calculated the among‐male coefficient of variation in total sperm length (CV_am_). These coefficients of variation were calculated as (*SD*/mean)*100*(1 + 1/(4*n*)), with the final component adjusting for small sample size (Sokal & Rohlf, 1995). One person took all sperm measurements. Although formal blinding of sample identity was not conducted, species identity was not explicitly linked to sperm slides, such that bias in measurements was unlikely to occur.

To assess the proportion of normal sperm, we scored 10–126 cells per male (mean ± *SD* 87.1 ± 36.6 cells per male) for 22 males (all 4 intermediates with sufficient sample size, all 9 saltmarsh males, and 9 randomly chosen Nelson's sparrow males). We aimed to score the first approximately 100 cells that were visible in their entirety from each male's sample, taking additional photographs from samples with sufficient sperm concentrations to facilitate blinding of samples (since we expected some cells to be insufficient for scoring, upon more detailed examination). We scored fewer cells only when too few fully visible cells were available. Photograph names were randomized, and photographs were shuffled across all individuals prior to scoring to ensure blind scoring. Abnormality criteria were based on du Plessis and Soley (2011), the World Health Organization (2010), and du Plessis et al. (2014). A preliminary scan of samples indicated that the most common, and most readily scored, abnormalities were as follows: acephaly (no head), macrocephaly (abnormally large head), malformation of the helical head shape, acute bending of the head, un‐coiling of the midpiece from the flagellum, having two or more tails, or having a coil or loop at the tail tip rather than a straight tail (Figure S1). Other categories of abnormality, such as retained cytoplasm, were not considered because it was not possible to reliably distinguish them from debris adhered to the cell (since confirmation via electron microscopy was beyond the scope of this study). For simplicity, analysis was on normal versus abnormal; a summary of the types of abnormalities is in Table S1. One person (GG) scored all cells analyzed for species‐level comparisons. Another observer (ERAC) scored 300 of the same cells to assess robustness of categorization. Both measurers found 12.3% abnormal cells (37/300), with scores for individual cells differing for 2.7% cells (8/300, with each observer scoring 4 cells as normal that the other scored as abnormal). We measured repeatability in normal status (normal versus. abnormal) for each cell using the rptBinary function (Stoffel et al., 2017), controlling for unique cell ID as a random effect. Repeatability was significant, with 98.7 ± 0.4 (*SE*)% of the variation attributable to cell identity (*p* < .001, 95% CI: 98.2–99.7).

### 2.4 Statistical analysis

We tested whether species differed in the likelihood that the fluid obtained by cloacal massage contained sperm, using a chi‐squared test.

As the clearest analysis of divergence between species, we compared the length for each sperm segment and total sperm length between species using separate linear mixed models (package lme4, Bates et al., 2015) for each sperm measure. Male identity was a random effect, and species category (three categories, based on the SNP hybrid index: saltmarsh and back‐crossed saltmarsh; Nelson's and back‐crossed Nelson's; and intermediates) was the only fixed effect. Species categories, however, encompass substantial variation in SNP hybrid index and in the (summed) plumage index. Specifically, plumage index and hybrid index are highly correlated across all individuals (*F*_1,40_ = 204.2, *p* < .001, adjusted *r* ^2^ = 0.83), but uncorrelated when each species category is analyzed individually (saltmarsh sparrows *F*_1,18_ = 0.211, *p* = .65, adjusted *r* ^2^ = −0.04; Nelson's sparrows *F*_1,15_ = 0.40, *p* = .53, adjusted *r* ^2^ = −0.03; intermediates *F*_1,3_ = 1.85, *p* = .27, adjusted *r* ^2^ = 0.18). We therefore test how total sperm length relates to plumage index, since selection acts at the phenotypic level, making it important to understand the phenotype as an integrated whole (Shaw & Mullen, 2011). We also test how sperm morphology relates to hybrid index, as this better reflects genetic ancestry. We constructed a separate mixed model for each of these predictors, with male identity as a random effect; including both predictors in the same model resulted in a variance inflation factor of 4.8, above the recommended threshold of 3 (Zuur et al., 2009). Significance was assessed using the package lmerTest with Satterthwaite's approximation of degrees of freedom (Kuznetsova et al., 2017), and the amount of variance explained was assessed by calculating the marginal *r* ^2^ (which reflects variance explained by fixed effects) and the conditional *r* ^2^ (which reflects variance explained by fixed and random effects) in package MuMIn (Barton, 2020). Model assumptions were assessed visually, as recommended by Zuur et al. (2009). Because these tests examined interdependent variables, we corrected *F* test results for multiple testing across these 6 analyses (total sperm length and each of the 3 sperm segments versus species category; total sperm length versus plumage index; and total sperm length versus hybrid index) using Bonferroni adjustment. Since qualitative results were unchanged, we present uncorrected p‐values.

In addition, we compared within‐male variation in total sperm length in a linear model with standard deviation in sperm length as the response, species as the predictor of interest, and mean total sperm length of the male as a covariate, to control for expected increased variation in larger measurements (Fitzpatrick & Baer, 2011). We further compared within‐species variance in total sperm length using a Levene's test (note that controlling for mean length was not feasible, and that the larger measurements tended to have lower variability in our dataset, making the Levene test conservative). We compared the proportion of normal sperm across categories using a generalized linear mixed model, with the proportion of normal sperm as the response variable, male identity as a random effect, and species category as a fixed effect, with a binomial link function. This approach takes into consideration variation in the number of cells scored across males. We compared this model to a model without the fixed effect of species with a likelihood ratio test.

Finally, we assessed whether the length of the head, midpiece, and tail was correlated. Here, we examined both within‐ and between‐individual patterns following the recommendation of van de Pol and Wright (2009). Specifically, we calculated the mean length of each segment, which represents between‐individual effects, and deviation from each sperm cell's measure to the mean, which represents within‐individual effects. We then constructed models with raw values for one segment as the response variable, male identity as a random effect, and fixed effects of the mean and deviation from the mean for other sperm segments, as well as species category as a covariate. All analyses were conducted in R v. 4.0.2 (R Development Core Team, 2021).

## 3 RESULTS

**FIGURE 1:**
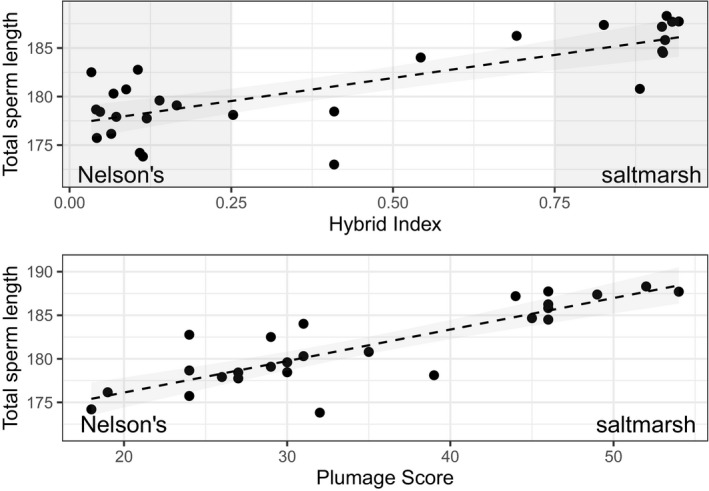
Total sperm length (µm) correlates with both (a) hybrid index based on 135 fixed SNPs and (b) plumage index based on 13 plumage traits. Data are from saltmarsh sparrow, Nelson's sparrow, and intermediate males captured at two sites in the hybrid zone; lower values indicate more Nelson's‐like, and higher values indicate more saltmarsh‐like, for hybrid index and plumage index. Gray shading in panel A shows regions defined as Nelson's sparrows (left) or saltmarsh sparrows (right). Shading of the dashed line indicates 95% confidence intervals. Plumage index was unavailable for two individuals

Of the 46 males that produced fluid following cloacal massage, 28 produced samples with at least one sperm cell (Table 1). Success at obtaining sperm varied across species, with intermediates being more likely than expected to produce sperm and both saltmarsh and Nelson's males being less likely ($\chi_{2}^{2}$ = 6.18, *p* = .05; Table 1). There was significant variation among the three species categories in total sperm length (*F*_2,23.3_ = 17.11, *p* < .001) and midpiece length (*F*_2,23.7_ = 18.29, *p* < .001) but not in sperm head length (*F*_2,25_._7_ = 1.23, *p* = .31), sperm tail length (*F*_2,22.9_ = 0.23, *p* = .80), or CV_wm_ (*F*_2,22_ = 2.31, *p* = .12; Table 1). The among‐category variation in length was driven by differences between saltmarsh males compared with Nelson's and intermediate males (post hoc test results in Table 1). Among‐male variation in total sperm length did not differ significantly between saltmarsh and Nelson's sparrows (*F*_1,21_ = 0.29, *p* = .60; intermediates not included in the test). Total sperm length correlated tightly with the hybrid index based on SNPs (*F*_1,23.0_ = 43.79, *p* < .001, marginal *r* ^2^ = 0.51; conditional *r* ^2^ = 0.79; Figure 1a) and the plumage index (*F*_1,19.4_ = 89.38, *p* < .001, marginal *r* ^2^ = 0.64, conditional *r* ^2^ = 0.79; Figure 1b). Species category did not explain significant variation in the proportion of sperm cells with normal morphology ($\chi_{2}^{2}$ = 2.32, *p* = .31; Table 1, Table S1). Rather, the percent normal sperm showed substantial variation within each category (from about 40%–60% normal to >95% normal within each category; Table S1).

**TABLE 1:** Sperm sampling and morphology in the hybrid zone between saltmarsh and Nelson's sparrows

Species (*N* success, *N* tried^a^)	CV_am_	Head	Midpiece	Tail	TSL	CV_wm_ ^b^	Proportion normal
Saltmarsh (9/21)	1.32	16.68 ± 0.48^A^	161.37 ± 2.50^A^	7.97 ± 0.78 ^A^	186.01 ± 2.39^A^	1.16 ± 0.27^A^	0.89 ± 0.13^A^
Nelson's (14/19)	1.57	16.90 ± 0.38^A^	153.26 ± 2.21^B^	8.25 ± 1.61 ^A^	178.40 ± 2.75^B^	1.32 ± 0.47^A^	0.83 ± 0.15^A^
Intermediate (5/6)	3.06	17.00 ± 0.20^A^	154.61 ± 6.20^B^	8.35 ± 1.80 ^A^	179.97 ± 5.25^B^	1.46 ± 1.23^A^	0.77 ± 0.24^A^

Note

This table shows the number of males for which cloacal massage produced fluid containing sperm (*N* success), out of number of males where it produced fluid (*N* tried); the length of sperm segments (mean ± *SD*, µm); coefficient of variation for total sperm length (TSL) among (CV_am_) and within (CV_wm_) males; and the proportion of sperm cells with normal morphology (assessed for 9 saltmarsh, 9 Nelson's, and 4 intermediate males). Post hoc comparisons of mean segment lengths were assessed by releveling reference values for traits with significant *F* tests, with significant differences indicated by different superscript letters (*p* < .05; differences robust to correction for multiple testing). Saltmarsh and Nelson's designations include both pure and back‐crossed individuals, while intermediates include F1 and F2 hybrids, with species assignment based on the hybrid index from 135 SNPs.

^a^ One saltmarsh and one Nelson's sparrow did not have genetic information and thus were identified on the basis of plumage only.

^b^ Two Nelson's and one intermediate male with <5 cells measured were excluded from calculating mean and *SD* for CV_wm_.

Sperm cells with longer midpieces had longer heads at the within‐individual level (*t*_228.6_ = 3.37, *p* < .001), but not at the among‐individual level (*t*_29_._7_ = 0.82, *p* = .42), as well as shorter tails at both levels (within; t_226.55_ = −4.75, *p* < .001, among: *t*_29.9_ = 2.20, *p* = .04). Head and tail lengths were not related at either level (|*t*|< 1.14, *p* > .25).

## 4 DISCUSSION

Saltmarsh sparrow sperm was approximately 4.4% longer than Nelson's sparrow sperm. This level of differentiation is typical for that observed between sister species (mean ± *SD*, 3.5 ± 4.4%, Hogner et al., 2013), and it corroborates the possibility that sperm may act as a prezygotic barrier between species. Total sperm length correlates with the length of female sperm storage tubules across species (Briskie et al., 1997), such that sperm may not be stored by the female effectively if it is not of the appropriate length for the species. Potential differences in the physiology of sperm or the female reproductive tract between species linked to the divergence in osmotic balance regimes between the saltmarsh specialist and the more generalist species (Walsh et al., 2019) are another possible source of sperm–female incompatibility, since sperm motility responds to factors such as calcium ion concentration and pH in other birds (Holm & Wishart, 1998; Wishart & Wilson, 1999). Examining whether female sperm storage tubules have diverged, and how sperm from each species performs in the reproductive tract fluid of the other species, would be interesting next steps. Previous experiments using the latter approach find reduced sperm swimming performance in fluid from the reproductive tract of heterospecific females in *Ficedula* flycatchers, where hybridization is ongoing and costly due to high postzygotic isolation (Cramer et al., 2016), but not in species pairs without interbreeding (Cramer et al., 2014; Cramer, Stensrud et al., 2016). Tests in this hybrid zone, where interbreeding occurs but postzygotic isolation may be lower, would be informative.

Both saltmarsh and Nelson's sparrow males showed low among‐male variation in total sperm length (CV_am_ = 1.32, 1.57; in other passerines, CV_am_ ranges from 1.07 to 9.62, mean ± *SD*, 2.91 ± 1.5, *n* = 129 species; Lifjeld et al., 2019) and within‐male variation (CV_wm_ = 1.16, 1.32; in other passerines, CV_wm_ ranges from 0.97 to 3.64, mean ± *SD*, 1.84 ± 0.63, *n* = 65 species; Lifjeld et al., 2010). Since these measures, and especially CV_am_, decrease across species with increasing multiple mating by females (Lifjeld et al., 2010), these low values are consistent with the exceptionally high levels of multiple paternity observed in these species. The lower CV_am_ saltmarsh sparrows than in Nelson's sparrows were expected since saltmarsh sparrows have higher rates of multiple paternity (Maxwell, 2018), perhaps because they do not guard females following copulation as Nelson's sparrow males do (Shriver et al., 2007). Furthermore, Saltmarsh sparrows have a larger cloacal protuberance than Nelson's sparrows (Maxwell, 2018), and variance in reproductive success appears to be higher in saltmarsh sparrow males (Maxwell, 2018; Walsh, Maxwell et al., 2018). Finally, saltmarsh sparrow sperm is longer than Nelson's sparrow sperm, and longer sperm length is associated with higher postcopulatory sexual selection across species (e.g., Rowe et al., 2015). Estimated CV_am_ may be somewhat inflated in this study due to introgression between species, and similarly, variation in the genetic make‐up of intermediate males may explain the relatively high CV_am_ in these males (CV_am_ = 3.06). If postcopulatory sexual selection is indeed higher for saltmarsh sparrows, their sperm may be expected to be highly successful competitors in both conspecific and heterospecific contexts, for example, due to faster swimming speed (Kleven et al., 2009), preferential access to female sperm storage tubules (as observed for longer, faster‐swimming sperm in zebra finches; Hemmings & Birkhead, 2017), or greater ability to penetrate ova (as observed in *Mus* species or laboratory populations with stronger postcopulatory sexual selection: Martín‐Coello et al., 2009; Firman et al., 2014). If such a mechanism is at work in this hybrid zone, sperm may act as an asymmetric rather than a bidirectional prezygotic barrier between species, with saltmarsh sparrow males having higher fertilization success.

In contrast to the potential for sperm to act as a prezygotic barrier, our preliminary evidence does not suggest that it is likely to act as a postzygotic barrier. Specifically, sperm morphology of intermediate males was similar to Nelson's sparrows, intermediate males did not show a higher proportion of abnormal sperm, and intermediate males are known to sire offspring (Maxwell, 2018; Walsh, Maxwell et al., 2018). In *Ficedula* flycatchers, hybrid males are infertile and do not produce normal sperm cells (Ålund et al., 2013), while in other hybridizing passerines with a similar time since divergence to flycatchers, hybrid sperm appear normal (Albrecht et al., 2019; Cramer et al., 2015). Samples collected from intermediate males in this study were also more likely to contain sperm than were samples from pure and back‐crossed individuals. While variation in sperm sampling success is not well‐understood, males that have not copulated recently (i.e., that have not depleted their sperm stores) might be more likely to produce sperm under cloacal massage. Intermediate males are hypothesized to be at a disadvantage in courtship, being too large for the aerial acrobatics typical of Nelson's sparrows and too small for successful scramble competition against saltmarsh sparrows (Walsh, Maxwell et al., 2018). Indeed, a high proportion of intermediate males sire no offspring (81% of 33 intermediate males, compared to 60% of 213 pure and back‐crossed males of both species in Walsh, Maxwell et al., 2018; or 53% of 17 intermediate males, compared to 25% of 103 pure and back‐crossed males of both species in Maxwell, 2018), although the number of offspring sired by successful intermediate males is similar to Nelson's sparrows (Maxwell, 2018; Walsh, Maxwell et al., 2018). Together, these observations may suggest that intermediate males suffer reduced copulation success but not reduced fertilization success following copulation, although disentangling pre‐ and postcopulatory processes using parentage data is challenging (Cramer, 2021).

For species with substantial sexual selection at both the pre‐ and postcopulatory stages, correlations between male traits promoting copulation success and sperm traits promoting fertilization success may have important consequences for trait evolution (Polak et al., 2021; Simmons et al., 2017), as well as for our ability to study it (Cramer, 2021). While this idea has been raised in many intraspecific studies, the relationships between pre‐ and postcopulatory traits that act as reproductive barriers are not well studied in hybrid zones. We found that sperm morphology correlated with plumage index, such that males with relatively saltmarsh‐like sperm also have relatively saltmarsh‐like plumage. This correlation may be simply a historical artifact of divergence between species during allopatry, in which case divergence in sperm length in allopatric populations is expected to be similar to divergence within the hybrid zone. The correlation may also have fitness consequences, if plumage acts as a signal of sperm phenotype and thereby allows females to obtain compatible sperm, similar to the phenotype‐linked sperm hypothesis within species (Sheldon, 1994). However, the role of plumage in assortative mating, and the degree of female control over copulation, is not fully known in this system (Greenlaw & Post, 2012). At a minimum, within‐species copulatory advantages would be accentuated by a conspecific sperm function advantage. Conversely, even without a postcopulatory prezygotic barrier, low copulation success of intermediate males would cause indirect selection against their sperm traits (although, preliminarily, intermediates do not have a sperm phenotype distinct from nonintermediates). With sufficiently strong postzygotic isolation, reinforcement could cause an accentuation in the divergence in plumage, sperm, or both in the hybrid zone, similar to the case in *Luscinia* nightingales where sperm head length is more diverged within the hybrid zone than outside it (Albrecht et al., 2019). However, without linkage among loci causing postzygotic isolation and phenotypic divergences, reinforcement acting on either plumage or sperm could actually slow the evolution of a reproductive barrier based on the other trait via reinforcement, because the presence of one barrier reduces the selective pressure promoting the other (Lorch & Servedio, 2007; Marshall et al., 2002). Understanding phenotypic and genetic correlations among different phenotypes relevant for reproductive isolation remains an important challenge in studying hybrid zones and may assist with understanding how reproductive barriers accumulate over evolutionary time, and how each individual reproductive barrier contributes to overall reproductive isolation (e.g., Larson et al., 2019; Mendelson et al., 2007).

Assortative offspring production in the *Ammospiza* hybrid zone has previously been noted (Maxwell, 2018; Walsh, Maxwell et al., 2018). Precopulatory behaviors likely play a role: Nelson's sparrow males sing and perform aerial displays, while saltmarsh sparrow males engage in scramble polygyny (Greenlaw, 1993; Maxwell, 2018; Shriver et al., 2007; Walsh, Maxwell et al., 2018). Here, we show that sperm morphology, like precopulatory phenotypes, has diverged between species, and we suggest that it may contribute to reproductive isolation and assortative offspring production. Sperm phenotypes largely correlate with plumage phenotypes, suggesting that these traits will evolve in concert with each other, rather than in opposition. We thus add a further layer of understanding of an exceptionally complex hybrid system, characterized by diverse sexual and natural selective pressures in an intricate environmental mosaic (Maxwell et al., 2021; Walsh et al., 2019; Walsh, Kovach et al., 2018; Walsh, Rowe et al., 2016).

## CONFLICT OF INTEREST

The authors declare that they have no conflict of interest.

## AUTHOR CONTRIBUTION

**Emily R. A. Cramer:** Formal analysis (equal); Writing‐original draft (lead); Writing‐review & editing (equal). **Gaute Grønstøl:** Data curation (lead); Investigation (equal); Writing‐review & editing (equal). **Logan Maxwell:** Formal analysis (equal); Investigation (equal); Writing‐review & editing (equal). **Adrienne I. Kovach:** Conceptualization (supporting); Funding acquisition (equal); Investigation (equal); Project administration (equal); Resources (equal); Writing‐review & editing (equal). **Jan T. Lifjeld:** Conceptualization (lead); Funding acquisition (equal); Project administration (equal); Resources (equal); Writing‐review & editing (equal).

## Supporting information

Fig S1

Table S1

## Data Availability

Data are available from the Dryad Digital Repository, https://doi.org/10.5061/dryad.cc2fqz662. Sperm samples have been accessioned to the University of Oslo Natural History Museum (accession numbers 91982–92027).
