# Supplementary material for: Sperm length divergence as a potential prezygotic barrier in a passerine hybrid zone
Source: Ecol Evol. 2021 Jun 16;11(14):9489–97. doi: 10.1002/ece3.7768 (PMC8293778; doi:10.1002/ece3.7768)
Supplement: Supplementary file 1 — Fig S1 [file ECE3-11-9489-s001.pdf]

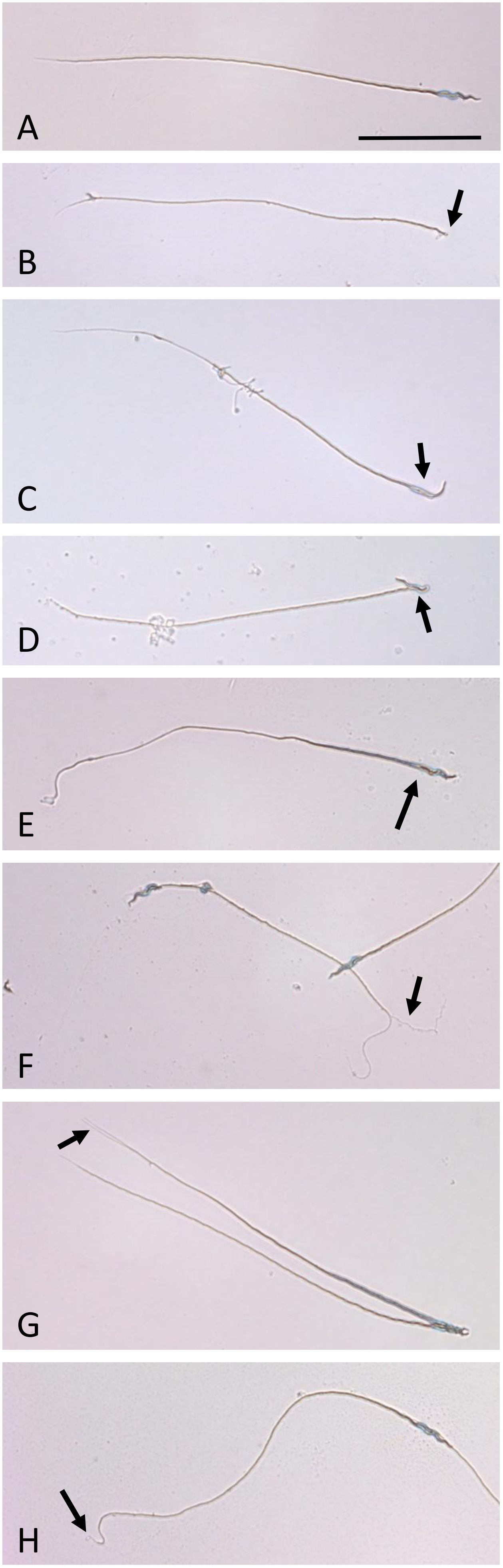

Figure S1. Example images of a normal sperm (A) and sperm abnormalities (B-H) scored in *Ammospiza* sparrows, with abnormality indicated by the arrow. Photos were taken at 320 X magnification; scale bar indicated in panel A is 50 µm and scale is the same across photos. B) acephaly. C) malformed head helix. D) Acute head bending. E) Macrocephaly. F) Uncoiling of the midpiece. G) double/split tail. H) tail loop/coil.
