# Supplementary material for: Sperm length divergence as a potential prezygotic barrier in a passerine hybrid zone
Source: Ecol Evol. 2021 Jun 16;11(14):9489–97. doi: 10.1002/ece3.7768 (PMC8293778; doi:10.1002/ece3.7768)
Supplement: Supplementary file 2 — Table S1 [file ECE3-11-9489-s002.docx]

| Table S1. Detailed summary of types of abnormalities in sperm cells of Nelson's sparrow, saltmarsh sparrow, and intermediate males. Data are sorted in increasing order of % normal sperm within species categories. | | | | | | | | | | | |
| --- | --- | --- | --- | --- | --- | --- | --- | --- | --- | --- | --- |
| Accession number | species | % Normal | N Normal | N scored | N abnormal, by abnormality type | | | | | | |
|  |  |  |  |  | Acephaly | Malformed head helix | Acute head bending | Macrocephaly | Uncoiling of midpiece | Double/split tail | Tail loop/coil |
| 92013 | Nelson's | 52.4 | 66 | 126 | 42 | 0 | 1 | 0 | 52 | 0 | 1 |
| 92019 | Nelson's | 71.9 | 46 | 64 | 10 | 1 | 0 | 4 | 9 | 0 | 1 |
| 92007 | Nelson's | 76.4 | 55 | 72 | 5 | 0 | 1 | 0 | 15 | 1 | 1 |
| 92008 | Nelson's | 78.9 | 75 | 95 | 7 | 1 | 0 | 0 | 18 | 0 | 1 |
| 92018 | Nelson's | 88.7 | 94 | 106 | 6 | 0 | 3 | 0 | 8 | 0 | 1 |
| 92014 | Nelson's | 89.3 | 75 | 84 | 2 | 0 | 0 | 0 | 8 | 1 | 0 |
| 92017 | Nelson's | 95.7 | 110 | 115 | 4 | 0 | 0 | 0 | 1 | 0 | 1 |
| 92012 | Nelson's | 96.5 | 110 | 114 | 0 | 0 | 0 | 0 | 0 | 0 | 4 |
| 92021 | Nelson's | 96.6 | 115 | 119 | 1 | 1 | 0 | 2 | 3 | 0 | 0 |
| 91993 | Saltmarsh | 60.6 | 20 | 33 | 7 | 4 | 0 | 4 | 4 | 0 | 1 |
| 92002 | Saltmarsh | 80.0 | 8 | 10 | 2 | 0 | 0 | 0 | 2 | 0 | 1 |
| 91983 | Saltmarsh | 85.5 | 100 | 117 | 6 | 1 | 1 | 6 | 1 | 1 | 3 |
| 91994 | Saltmarsh | 88.9 | 48 | 54 | 1 | 0 | 1 | 0 | 3 | 0 | 0 |
| 91988 | Saltmarsh | 91.7 | 22 | 24 | 0 | 0 | 0 | 0 | 1 | 0 | 1 |
| 91996 | Saltmarsh | 94.7 | 107 | 113 | 0 | 0 | 0 | 0 | 2 | 0 | 4 |
| 91985 | Saltmarsh | 97.4 | 114 | 117 | 1 | 1 | 0 | 0 | 1 | 0 | 1 |
| 91987 | Saltmarsh | 100.0 | 116 | 116 | 0 | 0 | 0 | 0 | 0 | 0 | 0 |
| 92027 | Saltmarsh | 100.0 | 121 | 121 | 0 | 0 | 0 | 0 | 0 | 0 | 0 |
| 91986 | Intermediate | 42.7 | 47 | 110 | 50 | 0 | 0 | 0 | 58 | 1 | 1 |
| 92023 | Intermediate | 79.0 | 79 | 100 | 10 | 1 | 2 | 0 | 5 | 0 | 7 |
| 92011 | Intermediate | 88.0 | 22 | 25 | 2 | 0 | 0 | 0 | 0 | 0 | 2 |
| 92026 | Intermediate | 98.8 | 81 | 82 | 1 | 0 | 0 | 0 | 0 | 0 | 0 |
